# Diagnostic accuracy of administrative data algorithms in the diagnosis of osteoarthritis: a systematic review

**DOI:** 10.1186/s12911-016-0319-y

**Published:** 2016-07-07

**Authors:** Swastina Shrestha, Amish J. Dave, Elena Losina, Jeffrey N. Katz

**Affiliations:** Department of Orthopedic Surgery, Orthopaedic and Arthritis Center for Outcomes Research, Brigham and Women’s Hospital, 75 Francis St, BC 4-016, Boston, MA 02115 USA; Harvard Medical School, Boston, MA USA; Division of Rheumatology, Immunology and Allergy, Brigham and Women’s Hospital, Boston, MA USA; Department of Epidemiology, Harvard School of Public Health, Boston, MA USA; Department of Biostatistics, Boston University School of Public Health, Boston, MA USA

**Keywords:** Osteoarthritis, Diagnostic accuracy, Administrative data, Systematic review

## Abstract

**Background:**

Administrative health care data are frequently used to study disease burden and treatment outcomes in many conditions including osteoarthritis (OA). OA is a chronic condition with significant disease burden affecting over 27 million adults in the US. There are few studies examining the performance of administrative data algorithms to diagnose OA. The purpose of this study is to perform a systematic review of administrative data algorithms for OA diagnosis; and, to evaluate the diagnostic characteristics of algorithms based on restrictiveness and reference standards.

**Methods:**

Two reviewers independently screened English-language articles published in Medline, Embase, PubMed, and Cochrane databases that used administrative data to identify OA cases. Each algorithm was classified as restrictive or less restrictive based on number and type of administrative codes required to satisfy the case definition. We recorded sensitivity and specificity of algorithms and calculated positive likelihood ratio (LR+) and positive predictive value (PPV) based on assumed OA prevalence of 0.1, 0.25, and 0.50.

**Results:**

The search identified 7 studies that used 13 algorithms. Of these 13 algorithms, 5 were classified as restrictive and 8 as less restrictive. Restrictive algorithms had lower median sensitivity and higher median specificity compared to less restrictive algorithms when reference standards were self-report and American college of Rheumatology (ACR) criteria. The algorithms compared to reference standard of physician diagnosis had higher sensitivity and specificity than those compared to self-reported diagnosis or ACR criteria.

**Conclusions:**

Restrictive algorithms are more specific for OA diagnosis and can be used to identify cases when false positives have higher costs e.g. interventional studies. Less restrictive algorithms are more sensitive and suited for studies that attempt to identify all cases e.g. screening programs.

**Electronic supplementary material:**

The online version of this article (doi:10.1186/s12911-016-0319-y) contains supplementary material, which is available to authorized users.

## Background

Administrative health care data are collected by health care providers, insurers, and governments for enrollment, reimbursement, and payment purposes [1, 2]. Sources of administrative data include physician billing databases, hospitalization discharge records, prescription drug records, private insurers, managed care plan data systems, Medicare, and Medicaid [2]. Administrative data are used increasingly in health services research as they tend to be less expensive than manual medical record review, available for large populations, and unaffected by recall or selection biases [1, 3, 4]. Researchers also use administrative health care data to identify patients for inclusion in study cohorts as these data provide a less costly approach to identifying subjects than screening in person or by phone [5].

Along with these advantages, however, administrative data have limitations, such as misclassification, which may jeopardize study results [3]. An international consortium of researchers and administrative health care data users has identified validation of administrative data coding as a research priority [6]. To strike a balance between the specificity and sensitivity of administrative data, investigators create algorithms, which typically involve ‘and’ and ‘or’ statements to focus on diagnosis or procedures of interest. The US Food and Drug Administration’s (FDA) Mini-Sentinel Initiative has highlighted the importance of understanding the validity of administrative data algorithms for identifying health outcomes of interest [7, 8]. The accuracy of algorithms for identifying cases with specific diagnoses depends on features of the database, condition, study population, and reference standard for confirming the diagnosis. Many of the studies that establish the accuracy of administrative data algorithms lack consistent methodology and reporting standards, making it difficult to compare the data accuracy across studies [3]. These issues are of concern to investigators and policy makers worldwide as many health systems across the globe are making increasing use of administrative data.

This study examines the accuracy of administrative health care data algorithms for identifying patients with osteoarthritis (OA). OA is associated with significant burden, affecting 27 million adults in the US and more than 150 million adults worldwide [9, 10]. Administrative data play an important role in research on disease burden, treatment outcomes, and quality improvement across a range of conditions including OA [11–15]. However the accuracy of administrative data for the diagnosis of OA has received sparse study. One systematic review reported the accuracy of administrative data-based diagnosis in a wide range of rheumatologic conditions but provided limited detailed information on OA [16]. The goal of the present study is to perform a systematic review of studies of administrative data algorithms to diagnose OA and to evaluate the diagnostic characteristics of these algorithms based on restrictiveness and reference standards.

## Methods

### Study identification

This systematic review was performed based on the Preferred Reporting Items for Systematic Reviews and Meta-analyses (PRISMA) guidelines [17]. A search of all titles available in Medline, Embase, Cochrane, and PubMed was conducted using the following major keywords: *administrative data, validation studies, and osteoarthritis* (Additional file 1: Table S1 for search strings) [18]. We carried out the search on January 2015 and two reviewers (AJD and SS) screened every reference to determine whether the study met the inclusion criteria. We also reviewed the bibliographies of relevant articles to identify articles that might have been missed by our initial search. The search was repeated to include references published from January 2015 through February 2016.

### Inclusion and exclusion criteria

We included English-language studies that reported both sensitivity and specificity of administrative data algorithms to identify cases of symptomatic OA by comparing the algorithm with a reference standard. If the studies presented 2 by 2 tables of positive and negative cases (based on a reference standard) crossed with positive and negative putative cases (based on an administrative data algorithm), we used the table to calculate sensitivity and specificity of the algorithm using the formulas below [19]. True positives were cases that were identified by both algorithm and gold standard and true negatives were cases that were not identified by both. False positives were cases that were identified by the algorithm but not the gold standard and false negatives were cases that were identified by the gold standard but not by the algorithm.

$$ Sensitivity=\frac{True\  positives}{True\  positives+ False\  negatives} $$

$$ Specificity=\frac{True\  negatives}{True\  negatives+ False\  positives} $$

Studies only reporting positive predictive value (PPV) without reporting 2 by 2 tables (or including sensitivity and specificity values) were excluded. If the algorithm classified OA positive cases as definite and possible, we calculated the sensitivity and specificity based on only the definite cases. In studies that evaluated diagnostic algorithms for OA in multiple anatomic locations (e.g. hip, knee, hand and combinations of these joints), algorithms that combined all anatomic locations of OA were preferentially selected. Algorithms that used only imaging as the reference standard were excluded due to the variability in OA imaging classification criteria and frequent occurrence of positive imaging findings in asymptomatic persons. We contacted the authors of studies that reported other diagnostic measures such as kappa value to obtain the crude 2 by 2 table data for computing the sensitivity and specificity of the algorithms. Discrepancies between reviewers regarding the reasons for abstract and study exclusions were resolved by consultation with senior coauthors (JNK and EL).

### Data abstraction and quality assessment

From the articles that met our inclusion criteria, we extracted information on: author, year of publication, country of study, administrative data source and setting, location of OA, cohort characteristics (age, gender, size), description of the algorithm (minimum number of outpatient, prescription, and hospitalization codes, use of diagnosis information entered in electronic medical record, and years of administrative data), reference standard, disease prevalence in the sample, algorithm and reference standard positive and negative cases, and performance characteristics of the algorithms (positive predictive value, sensitivity and specificity with 95 % confidence intervals). When 95 % confidence intervals were not provided, we calculated them using the binomial distribution when possible. We considered OA diagnosis in the medical record as a proxy for physician diagnosis. For quality assessment of all included studies, we used the 40 point modified Standards for Reporting of studies of Diagnostic Accuracy (STARD) criteria [3]. If the study results were in abstract form prior to manuscript submission, we contacted the author for quality assessment of the study. The two reviewers (AJD and SS) independently completed all screening, data extraction, and quality reporting activities.

### Analysis

We classified the algorithms as restrictive or less restrictive based on the number and use of stringent codes such as procedural, hospitalization, or prescription codes to ascertain the diagnosis of OA. The algorithm was classified as restrictive if it required more than one code of any kind *or* if it required one or more stringent code such as procedural, prescription, or hospitalization codes. For example, each of the following algorithms would be classified as restrictive 1) an algorithm that required OA codes from two separate outpatient visits; 2) an algorithm that required one code from an outpatient visit *and* one prescription code; and 3) an algorithm that required a single hospitalization visit. Algorithms that only required a single OA code from one outpatient visit were classified as less restrictive. Additionally, an algorithm that required a single OA code from one outpatient visit *or* one prescription record would be deemed less restrictive because the more stringent prescription code was not *required* to identify OA diagnosis.

We recorded the sensitivity and specificity of all the OA ascertainment algorithms. For studies that did not report sensitivity and/or specificity, we calculated these values from 2 by 2 tables that stratified the sample based on algorithm positivity and reference standard positivity. We calculated the positive likelihood ratio (LR+) of the algorithms using the formula [20]:

$$ Positive\  likelihood\  ratio\ \left(LR+\right) = \frac{Sensitivity\ }{1- Specificity} $$

In order to calculate the positive likelihood ratios of algorithms with perfect specificity, we used the lower end of the confidence interval of specificity. Additionally we calculated positive predictive values (PPV) for different OA prevalence rates in order to highlight the prevalence dependence of the algorithm PPVs [21]. The PPV of an algorithm determines the probability that an individual identified by the algorithm truly has OA. We used the hypothetical proportion of 0.1 to approximate OA prevalence in general population, 0.25 to approximate OA prevalence in adults over 65, and 0.5 to approximate OA prevalence in specialty clinic settings [9, 12, 22].

## Results

### Search results

**Fig. 1:**
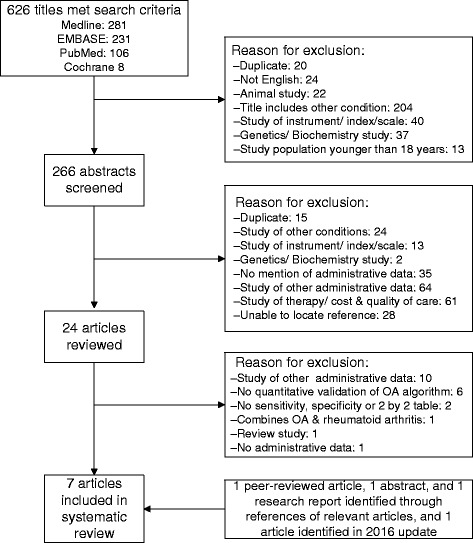
Study search and selection process

Our search strategy identified 626 unique articles. Upon screening the titles, we identified 266 articles for abstract review. 24 % (64/266) of abstracts were excluded because they addressed other administrative data; 23 % (61/266) were studies of quality of care, therapy, and cost-effectiveness; and 13 % (35/266) used no administrative data. We identified 24 references for full article review. Of these fully reviewed articles, 10 studied other administrative data, 6 did not include quantitative validation of the algorithm, 2 only reported the PPV of the algorithms but not sensitivity or specificity, 1 was a review, 1 combined codes for OA and rheumatoid arthritis, and 1 study compared self-reported OA diagnosis with medical records. We included 3 articles from this search in our final analysis. In addition, we identified 1 peer-reviewed article, 1 abstract, and 1 research report from searching the bibliographies of relevant articles. The updated search on February 2016 identified 1 eligible article, which was included in the review. Figure 1 outlines the study selection process.

### Features of abstracted studies

Table 1 describes the characteristics of the 7 included studies. Study sample size ranged from 171 to 5589 and sources of administrative data included Medicare claims, health maintenance organizations (HMO), primary care surveillance network, and health data repositories. Five studies were published in peer-reviewed journals [23–27], one was published as a research report [28], and one as an abstract [29]. The reference standards for positive OA diagnosis were self-report, American College of Rheumatology (ACR) classification criteria for OA, and physician diagnosis. One study compared the diagnostic accuracy of algorithms using multiple reference standards, including plain radiograph, MRI, self-report, and ACR classification criteria. 13 algorithms from these 7 studies were included in the final analysis, of which 5 were classified as restrictive and 8 were classified as less restrictive.

**Table 1:** Characteristics of included studies

Study	Country	Sample Size	Diagnosis	Age description	% Female	Admin Data source	Study Population
Fowles et al. 1995 [23]	US	1596	Unspecified OA	65 and above	Not reported	Medicine Parts A and B claims	Primary care patients in Maryland
Harrold et al. 2000 [25]	US	599	Unspecified OA	18 and above	62 %	Health maintenance organization (HMO)	Multispecialty group practice patients
Lix et al. 2006 [28]	Canada	5589	Unspecified OA	19 and above	Not reported	Population Health Research Data Respiratory	General Manitoba population
Rahman et al. 2008 [29]	Canada	171	Knee OA	Range 40–79	Not reported	BC Linked Health Database	Subjects with knee pain from a population based study of OA
Kadhim-Saleh et al. 2013 [24]	Canada	313	Unspecified OA	Mean age 68	52 %	Canadian Primary Care Sentinel Surveillance Network	Ontario Primary care research network
Williamson et al. 2014 [26]	Canada	1920	Unspecified OA	85 % above 60	55.5 %	Canadian Primary Care Sentinel Surveillance Network	Primary care research network in Canada
Coleman et al. 2015 [27]	Canada	403	Unspecified OA	90 % above 60	67 %	Canadian Primary Care Sentinel Surveillance Network	Mantibo Primary care research network

### Performance characteristics stratified by reference standard type

**Fig. 2:**
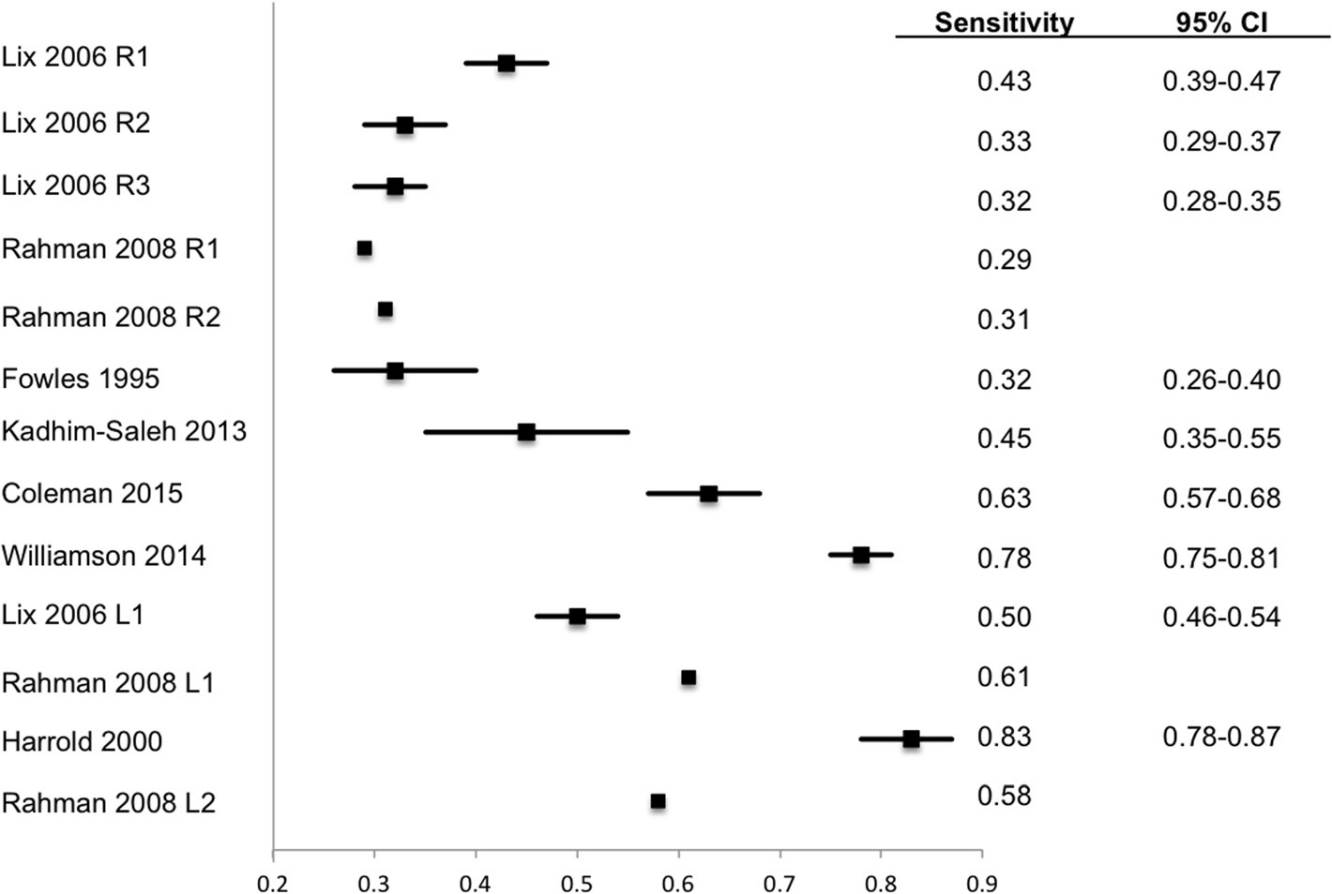
Forest plot of sensitivity of OA diagnosis algorithms. Table 2 provides description of each algorithm Error bars show 95 % confidence intervals (CI). Error bars are missing for Rahman 2008 as they did not report CI. Lix 2006 R1: hospitalization OR 2 physician visits OR 1 physician visit and Rx ICD-9-CM diagnostic codes in 5 years Lix 2006 R2: 1 hospitalization OR 2 physician visits ICD-9-CM diagnostic codes in 5 years Lix 2006 R3: 2 physician visits ICD-9-CM diagnostic codes in 5 years Rahman 2008 R1: 2 physician visits in 2 years OR 1 hospitalization ICD-9-CM diagnostic code compared with self report Rahman 2008 R2: 2 physician visits in 2 years OR 1 hospitalization ICD-9-CM diagnostic code compared with ACR criteria Lix 2006 L1: 1 physician visit ICD-9-CM diagnostic code in 5 years Rahman 2008 L1: 1 physician visit or 1 hospitalization ICD-9-CM diagnostic code compared with self report Rahman 2008 L2: 1 physician visit or 1 hospitalization ICD-9-CM diagnostic code compared with ACR criteria

**Fig. 3:**
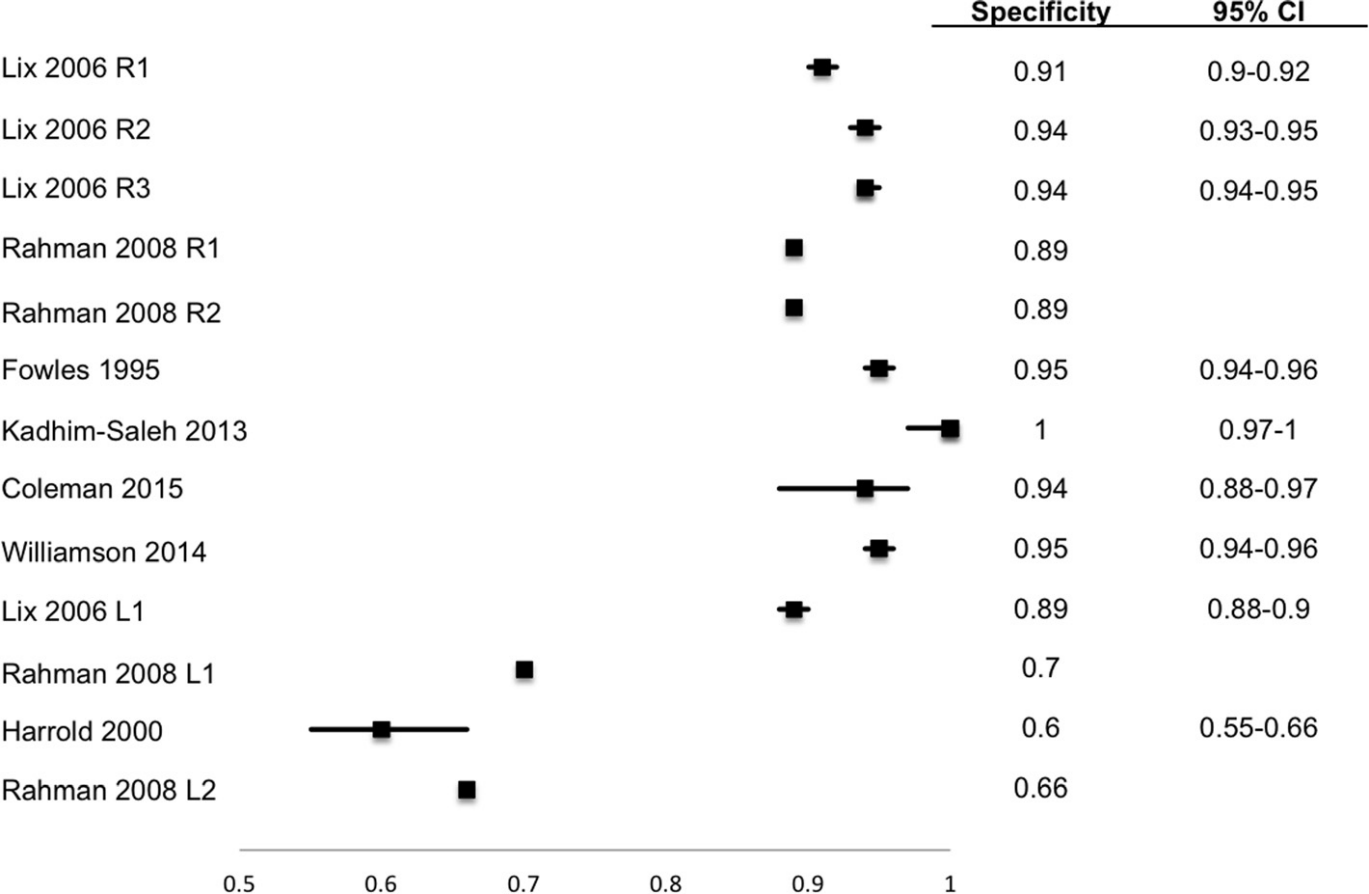
Forest plot of sensitivity of OA diagnosis algorithms. Table 2 provides description of each algorithm. Error bars show 95 % confidence intervals (CI). Error bars are missing for Rahman 2008 as they did not report CI. Lix 2006 R1: 1 hospitalization OR 2 physician visits OR 1 physician visit and 2 Rx ICD-9CM diagnostic codes in 5 years Lix 2006 R2: 1 hospitalization OR 2 physician visits ICD-9-CM diagnostic codes in 5 years Lix 2006 R3: 2 physician visits ICD-9-CM diagnostic codes in 5 years Rahman 2008 R1: 2 physician visits in 2 years OR 1 hospitalization ICD-9-CM diagnostic code compared with self report Rahman 2008 R2: 2 physician visits in 2 years OR 1 hospitalization ICD-9-CM diagnostic code compared with ACR criteria Lix 2006 L1: 1 physician visit ICD-9-CM diagnostic code in 5 years Rahman 2008 L1: 1 physician visit or 1 hospitalization ICD-9-CM diagnostic code compared with self report Rahman 2008 L2: 1 physician visit or 1 hospitalization ICD-9-CM diagnostic code compared with ACR criteria

The sensitivity, specificity, LR+, and PPV at assumed prevalence values of 0.1, 0.25, and 0.5 of individual algorithms are shown in Table 2. Table 3 reports the same diagnostic performance characteristics aggregated across restrictive versus less restrictive algorithms and across types of reference standard. The sensitivity and specificity of the algorithms with 95 % CI is shown as forest plots in Figs. 2 and 3 respectively.

**Table 2:** Descriptive and diagnostic characteristics of administrative data algorithms

Algorithm restrictiveness	Refence standard	Study	Algorithm definition	Years spanned by admin data	Sensitivity		Specificity		Positive likelihood ratio	Calculated PPV at 10 % prevalence	Calculated PPV at 25 % prevalence	Calculated PPV at 50 % prevalence
						95 % CI		95 % CI				
Restrictive		Lix 2006 [28]	1 hospitalization OR 2 physician visits OR 1 physician visit and 2 Rx ICD-9-CM diagnostic codes in 5 years	5	0.43	0.39–0.47	0.91	0.90–0.92	4.63	0.35	0.61	0.83
		Lix 2006 [28]	1 hospitalization OR 2 physician visits ICD–9–CM diagnostic codes in 5 years	5	0.33	0.29–0.37	0.94	0.93–0.95	5.47	0.38	0.65	0.85
		Lix 2006 [28]	2 physician visits ICD–9–CM diagnostic codes in 5 years	5	0.32	0.28–0.35	0.94	0.94–0.95	5.54		0.64	0.84
	Self-report	Rahman 2008 [29]	2 physician visits in 2 years OR 1 hospitalization ICD-9-CM diagnostic code	2^b^	0.29		0.89		2.64	0.23	0.47	0.73
	ACR criteria	Rahman 2008 [29]	2 physician visits in 2 years OR 1 hospitalization ICD-9-CM diagnostic code	2^b^	0.31		0.89		2.82	0.24	0.48	0.74
Less restrictive	Medical Record Review	Fowles 1995 [23]	1 physician visit ICD-9-CM diagnostic code	1	0.32	0.26–0.40	0.95	0.94–0.96	6.40	0.42	0.68	0.86
		Kadhim–Saleh 2013^a^ [24]	1 ICD-9-CM diagnostic code OR problems list in EMR	unspecified	0.45	0.35–0.55	1 (0.97^δ^)	0.97–1.00	15.00	0.63	0.83	0.94
		Coleman 2015^a^ [27]	1 ICD-9-CM diagnostic code OR problems list in EMR	unspecified	0.63	0.57–0.68	0.94	0.88–0.97	10.50	0.54	0.78	0.91
		Williamson 2014^a^ [26]	1 ICD-9-CM diagnostic code OR problems list in EMR	unspecified	0.78	0.75–0.81	0.95	0.94–0.96	15.25	0.63	0.84	0.94
	Self-report	Lix 2006 [28]	1 physician visit ICD-9-CM diagnostic code in 5 years	5	0.50	0.46–0.54	0.89	0.88–0.90	4.42	0.34	0.60	0.82
	ACR criteria	Rahman 2008 [29]	1 physician visit or 1 hospitalization ICD-9-CM diagnostic code	2^b^	0.61		0.70		2.03	0.18	0.40	0.67
		Harrold 2000 [25]	1 inpatient or outpatient ICD-9-CM diagnostic code	3	0.83	0.78–0.87	0.60	0.55–0.66	2.10	0.19	0.41	0.67
		Rahman 2008 [29]	1 physician visit or 1 hospitalization ICD-9-CM diagnostic code	2^b^	0.58		0.66		1.71	0.16	0.36	0.63

^a^Case definitions are developed in EMR based database

^b^Visit codes were restricted to 2 years and timespan of hospitalization code was unspecified. Rahman 2008 did not report 95 % CI

^δ^Lower confidence interval of specificity (instead of 1) was used to calculate LR+s and PPVs

**Table 3:** Medians and ranges of diagnostic characteristics of administrative data algorithms

Algorithm restrictiveness	Reference standard	No of algorithms	Median sensitivity	Sensitivity range	Median specificity	Specificity range	Median positive likelihood ratio	Positive likelihood ratio range	Median PPV at 10 % prevalence	Median PPV at 25 % prevalence	Median PPV at 50 % prevalence
Restrictive	Self-report	4	0.33	0.29–0.43	0.92	0.89–0.94	5.05	2.64–5.50	0.36	0.62	0.84
	ACR criteria	1	0.31	NA	0.89	NA	2.82	NA	0.24	0.48	0.74
Less restrictive	Physician diagnosis	4	0.54	0.32–0.78	0.95	0.94–1.00	12.75	6.40–15.25	0.58	0.80	0.92
	Self-report	2	0.55	0.50–0.61	0.79	0.70–0.89	3.23	2.03–4.42	0.26	0.50	0.74
	ACR criteria	2	0.71	0.58–0.83	0.63	0.60–0.66	1.91	1.71–2.10	0.18	0.39	0.65

### Performance characteristics stratified by reference standard type

#### Self-report

The four assessments of restrictive algorithms with reference standard of self-report had lower sensitivity (median 0.33) and higher specificity (median 0.92) compared to two assessments of less restrictive algorithms (median sensitivity 0.55) and (median specificity 0.92). The restrictive algorithms had higher LR+ and PPVs compared to less restrictive algorithms (Table 3).

#### ACR criteria

The one assessment of restrictive algorithms with reference standard of ACR criteria had lower sensitivity (0.31) and higher specificity (0.92) compared to two assessments of less restrictive algorithms (median sensitivity 0.71) and (median specificity = 0.63).

#### Physician diagnosis

All the algorithms that had reference standard of physician diagnosis were less restrictive. Among these, 3 studies used EMR based algorithms and 1 study used a non-EMR based algorithm. The EMR based algorithms were highly specific (0.95) and modestly sensitive (0.63) and LR+ of EMR based algorithms ranged from 10.5 to 15.25. The non-EMR based algorithm was highly specific (0.95) but less sensitive (0.32) and LR+ of non-EMR based algorithm was 6.40.

#### Quality assessment

Table 4 shows the number of studies that met each of the data quality and reporting criteria (modified STARD criteria). All studies reported the type of study and location, described patient sampling, details of data collection, disease classification, methods of calculating accuracy, and discussed the applicability of findings. Most studies provided the age of the cohort, identified the diagnosis of the validation cohort, and described the inclusion and exclusion criteria. Only one study reported the severity of disease, 2 studies provided flow charts and no study revalidated the algorithm in a different population. The most commonly reported study statistics were positive predictive value (*n* = 6), sensitivity (*n* = 5), specificity (*n* = 5), and negative predictive value (*n* = 3). Of these, 6 studies provided the 95 % confidence interval for all reported diagnostic measures. Only 1 study reported the likelihood ratio and 2 studies calculated the prevalence of OA in the study population.

**Table 4:** Number of studies meeting individual STARD modified criteria for validating health administrative data

	Reported/Total
TITLE, KEYWORDS, ABSTRACT	
Identify article as study of assessing diagnostic accuracy	7/7
Identify article as study of administrative data	7/7
INTRODUCTION:	
State disease identification & validation one of goals of study	7/7
METHODS:	
*Participants in validation cohort:*	
Describe validation cohort (Cohort of patients to which reference standard was applied)	7/7
Age	6/7
Disease	6/7
Severity	1/7
Location/Jurisdiction	7/7
Describe recruitment procedure of validation cohort	6/7
Inclusion criteria	6/7
Exclusion criteria	6/7
Describe patient sampling (random, consecutive, all, etc.)	7/7
Describe data collection	7/7
Who identified patients and did selection adhere to patient recruitment criteria	5/7
Who collected data	6/7
*A priori* data collection form	6/7
Disease classification	7/7
Split sample (i.e. re-validation using a separate cohort)	0/7
*Test Methods:*	
Describe number, training and expertise of persons reading reference standard	6/7
If >1 person reading reference standard, quote measure of consistency (e.g. kappa)	6/7
Blinding of interpreters of reference standard to results of classification by administrative data e.g. Chart abstractor blinded to how that chart was coded	6/7
*Statistical Methods:*	
Describe methods of calculating/comparing diagnostic accuracy	7/7
RESULTS:	
*Participants:*	
Report when study done, start/end dates of enrollment	4/7
Describe number of people who satisfied inclusion/exclusion criteria	6/7
Study flow diagram	2/7
*Test results:*	
Report distribution of disease severity	1/7
Report cross-tabulation of index tests by results of reference standard	7/7
*Estimates:*	
Report at least 4 estimates of diagnostic accuracy	5/7
Diagnostic Accuracy Measures Resported:	
Sensitivity	5/7
Spec	5/7
PPV	6/7
NPV	4/7
Likelihood ratios	1/7
kappa	4/7
Area under the ROC curve/C-statistic	0/7
Accuracy/agreement	1/7
Report accuracy for subgroups (e.g. age, geography, differen sex, etc.)	2/7
If PPV/NPV reported, ratio of cases/controls of validation cohort approximate prevalence of condition in the population	2/7
Report 95 % confidence intervals for each diagnostic measure	5/7
DISCUSSION:	
Discuss the applicability of the validation findings	7/7

## Discussion

We conducted a systematic literature review of diagnostic accuracy studies of administrative data algorithms for osteoarthritis diagnosis and compared their accuracy based on restrictiveness and reference standards employed in the studies. More restrictive algorithms had lower sensitivities and higher specificities compared to less restrictive algorithms when the reference standards were ACR criteria and self-report. All the algorithms that were validated against physician diagnosis were less restrictive and had very high specificities. The high positive likelihood ratios in this group was driven by studies that validated OA diagnosis in the electronic medical record (EMR) based primary care database, Canadian Primary Care Sentinel Surveillance Network (CPCSSN), designed for chronic disease surveillance [30]. The database combined billing ICD-9 codes with information from the EMR that allowed for more rigorous case definitions.

Widdifield et al. conducted a systematic review of studies that validated administrative data algorithms to identify rheumatic diseases [16]. They included osteoarthritis among the conditions studied but did not provide any analyses of the performance characteristics of OA algorithms. The authors reported high variability in patient sampling, reference standards, and measures of diagnostic accuracy among studies [16]. They found that use of pharmaceutical codes across the range of rheumatic conditions increased algorithm specificity slightly but compromised sensitivity; we observed similar patterns in studies of OA [16]. Our study included five additional cohorts not included in Widdifield et al., and excluded 3 OA studies that did not provide adequate data to calculate likelihood ratios [16]. These differences notwithstanding, the two studies concurred in finding that greater restrictiveness increased specificity of the administrative data algorithm. Widdifield and colleagues also suggested that study algorithms using self-report as the reference standard had lower sensitivity compared to studies that used medical record review as the reference standard [16]. Our study found that the algorithms had similar sensitivity when the reference standard was self-reported diagnosis (0.55) compared to physician diagnosis in the medical record (0.54).

We calculated the positive likelihood ratio (LR+) and positive predictive values (PPV) of each algorithm at assumed prevalence rates of 0.1, 0.25, and 0.5. Many validation studies of administrative data algorithms only report PPV. However, the sensitivity and specificity of the algorithm are generally not influenced by disease prevalence [31], the PPVs depend on the underlying prevalence of the condition in the study population [21]. Our results show that for the same algorithm, the PPV improves when the underlying OA prevalence increases from 0.10 to 0.25 and 0.50. This suggests that when studies report high PPV, we cannot ascertain whether the high PPV stems from a good algorithm or the underlying high OA prevalence in the study sample. Therefore, qualification of the algorithm solely based on PPV may be misleading. Thus, the underlying OA prevalence of the study sample needs to be clearly specified to evaluate the PPV of administrative data algorithms.

OA is a common comorbidity in the older population and has been frequently cited as an underreported diagnosis in studies that use administrative data to identify medical conditions [4]. The performance characteristics of administrative data algorithms diagnosing OA were influenced by reference standard and algorithm restrictiveness. We found that most of the algorithms that identify OA are relatively insensitive, potentially missing about 55 % of the cases [23–29]. Several reasons could account for the low sensitivity. For example, the physician might record OA as a secondary diagnosis but not enter the billing code, choosing instead to focus on the primary diagnosis. This situation might arise when the primary diagnosis is semi urgent such as active coronary heart disease with congestive heart failure, physicians may not be inclined to code for OA in such a circumstance. It has been shown that when physicians see patients for more pressing problems they often do not code less pressing problems [32]. The specificity of the algorithms was relatively high and algorithms that were validated against physician diagnosis had the highest specificity. As a result, the likelihood ratios of the algorithms with physician diagnosis as the reference standard were very high. The specificity of algorithms that validated the diagnosis against ACR criteria might have been lower because ACR classification criteria for OA are stringent and not widely used in clinical settings to diagnose OA.

The restrictive algorithms had lower sensitivity and higher specificity compared to the less restrictive algorithms. Therefore, when the purpose of the algorithm is to identify and recruit a patient cohort for a research study such as a treatment trial, it is crucial that each subject has the disease in question. Thus, restrictive algorithms with high specificity are most useful. However, if the aim is to identify all positive cases of OA, such as a screening program, less restrictive algorithms with high sensitivity may be more useful -- especially if a second, more specific can be applied to those that screen positive on the algorithm in order to reduce the number of false positive cases.

Limitations of this review include the exclusion of studies written in languages other than English. We did not report Youden index of the algorithms as only one study reported this statistic. We did not include studies that reported only Kappa values, as we lacked the information to compute sensitivity and specificity for these algorithms. We did not include algorithms with radiographs as a reference standard as radiographs can be both insensitive and non-specific in persons with OA [33–35]. As a consequence diagnoses made on the basis of radiographic findings may be inaccurate. Such misclassification would bias findings of this review to the null. Also, we did not conduct a meta-analysis of the diagnostic accuracies due to substantial heterogeneity in the methodologies of the included studies. We did not select algorithms based on site of OA, as majority of the studies did not specify the site of OA. The studies were heterogeneous with respect to population characteristics (e.g. age range), settings (e.g. primary care, specialty clinics), and administrative data sources (e.g. Medicare, health maintenance organization, primary care surveillance database, and state database). These differences enhance generalizability of findings but the heterogeneity precludes formal quantitative synthesis of the study findings. Finally, we recognize that each of the reference standards used in these studies (self-report, physician diagnosis, ACR criteria) has advantages and drawbacks. The observation that restrictive algorithms were less sensitive and more specific across multiple reference standards supports the robustness of this finding.

## Conclusions

Administrative data algorithms with restrictive case definitions are more specific for the diagnosis of OA whereas algorithms with less restrictive case definition are more sensitive. In general, published algorithms designed to identify positive OA cases have low sensitivity, missing more than half the cases. Algorithms assessed with reference standard of physician diagnosis have higher sensitivity and specificity than algorithms assessed with reference of self-reported diagnosis or ACR criteria. Our assessment of article quality revealed variable and sparse reporting of several key methodological features such as OA severity and OA prevalence in the underlying population.

Our work has implications for research and policy. From a research standpoint, the most appropriate algorithm for a particular study will depend on whether the study would best be served by optimizing sensitivity (missing as few cases as possible) or optimizing positive predictive value (increasing the likelihood that a person characterized by the algorithm as having OA indeed has OA). Our data suggest that requiring more than one OA outpatient code or a specialized code (e.g. a pharmacy or a hospitalization claim) will increase specificity and PPV, whereas requiring simply a single outpatient OA code will enhance sensitivity at the expense of specificity. From a policy standpoint, in circumstances that employ administrative data to portray burden of disease without actually intervening in individuals, the overall level of misclassification may be the most relevant parameter as the goal would be to have as accurate a count as possible. If an algorithm is used to target a subgroup of patients for a specific intervention (such as a prevention or education program), an algorithm with high PPV may be the best approach to ensure that program resources are spent on persons who indeed have OA.

## Abbreviations

ACR, American College of Rheumatology; FDA, US Food and Drug Administration’s; LR+, Positive likelihood ratio; OA, Osteoarthritis; PPV, Positive predictive value; PRISMA, Preferred Reporting Items for Systematic Reviews and Meta-analyses; STARD, Standards for Reporting of studies of Diagnostic Accuracy

## Additional file

Addition file 1: Table S1. MEDLINE, EMBASE, COCHRANE AND PUBMED Search strategies; List of search strings and keywords used to identify studies in different databases. (DOCX 182 kb)
