# Supplementary material for: Diagnostic accuracy of administrative data algorithms in the diagnosis of osteoarthritis: a systematic review
Source: BMC Med Inform Decis Mak. 2016 Jul 7;16:82. doi: 10.1186/s12911-016-0319-y (PMC4936018; doi:10.1186/s12911-016-0319-y)
Supplement: Supplementary file 1 [file 12911_2016_319_MOESM1_ESM.docx]

Supplementary Table 1. MEDLINE, EMBASE, COCHRANE AND PUBMED Search strategies

MEDLINE
Database: Ovid MEDLINE(R) 1946 to November Week 3 2014, Ovid MEDLINE(R) Daily Update Novemb
26,2015

Search Strategy:
----
1 administrative data.ti,ab. (4116)
2 administrative database*.ti,ab. (2371)
3 Databases, Factual/ (45139)
4 factual database/ (45139)
5 Databases as Topic/ (8060)
6 database/ (0)
7 Medical Record Linkage/ (3616)
8 administrative databank*.ti,ab. (2)
9 factual database*.ti,ab. (16)
10 factual databank*.ti,ab. (2)
11 factual data.ti,ab. (73)
12 exp medical records/ (87508)
13 exp medical record/ ((87508)
14 exp medical records systems, computerized/ (26087)
15 (medical record or health record or medical records or health records).ti,ab. (69847)
16 medical transcription*.ti,ab. (82)
17 exp Registries/ (59003)
18 registry/ (55285)
19 (registry or registries).ti,ab. (65590)
20 (utilization data* or utilisation data* or claims data* or managed care data* or physician billing data* or h
21 (administrative healthcare data* or administrative health care data* or administrative health data* or adr
22 (medical records based index or claims based index).ti,ab. (6)
23 (register and (link or links or linked or linkage or linking)).ti,ab. (2725)
24 or/1-23 [ADMINISTRATIVE DATA (BROAD)] (292078)
